# Estimating the economic costs of skin cancer in New South Wales, Australia

**DOI:** 10.1186/s12889-015-2267-3

**Published:** 2015-09-23

**Authors:** Christopher M. Doran, Rod Ling, Joshua Byrnes, Melanie Crane, Andrew Searles, Donna Perez, Anthony Shakeshaft

**Affiliations:** Hunter Medical Research Institute, University of Newcastle, Locked Bag 1000, New Lambton, NSW 2305 Australia; Centre for Applied Health Economics, Griffith University, Logan Campus L03 2.15, Meadowbrook, 4131 Australia; Cancer Institute NSW, PO Box 41, Alexandria, NSW 1435 Australia; School of Public Health, University of Sydney, Level 6, The Hub, Charles Perkins Centre (D17), Sydney, NSW 2006 Australia; National Drug and Alcohol Research Centre, University of New South Wales Sydney, Sydney, NSW 2052 Australia

**Keywords:** Economic, Cost, Skin cancer, Epidemiology, New South Wales, Australia

## Abstract

**Background:**

Skin cancer is one of the most common cancers in the world. The increased incidence of skin cancer, combined with limited health care resources and tight budgetary conditions, has increased the importance of understanding the economic impact of skin cancer. This research estimates the economic cost of skin cancer in the Australian state of New South Wales.

**Method:**

An incidence based approach is used to estimate lifetime costs of skin cancer. Both direct and indirect costs are considered - direct costs include resources associated with the management of skin cancer and indirect costs refer to productivity costs associated with morbidity and premature mortality. Diagnosis of skin cancer was determined according to ICD-10 codes using principal diagnosis. Linked administrative data and regression modelling are used to calculate costs; presented as Australian dollars for the year 2010. The human capital approach is used to value present and future productivity losses.

**Results:**

The lifetime cost of the 150,000 incident cases of skin cancer diagnosed in NSW in 2010 is estimated at $536 million ($44,796 per melanoma and $2459 per non-melanoma). Direct costs accounted for 72 % of costs ($10,230 per melanoma and $2336 per non-melanoma) and indirect costs accounted for 28 % of costs ($34,567 per melanoma and $123 per non-melanoma). Direct costs are, on average, higher for females than males with indirect costs, on average, higher for males than females.

**Conclusion:**

This research provides new evidence on the economic cost of skin cancer and provides policy makers with information of the potential monetary savings that may arise from efforts to reduce the incidence of skin cancer.

## Background

Skin cancer is one of the most common cancers in the world [1–4]. The majority of skin cancers develop from exposure to ultraviolet radiation (UVR), particularly from sun exposure. The highest incidence rates of skin cancer worldwide are in Australia and New Zealand, where two out of every three people are likely to be diagnosed in their lifetime [5].

Basal cell carcinoma (BCC) is the most common form of skin cancer followed by squamous cell carcinoma (SCC) [1, 2]. Together, BCC and SCC make up the majority of non-melanoma skin cancers (NMSC). Malignant melanoma accounts for less than five percent of skin cancer cases, yet it represents the vast majority of skin cancer deaths in Australia [6]. The incidence of skin cancer is increasing in Australia, and the incidence rate is greater than breast, prostate, lung and colon cancers combined. In terms of prevalence, more people have been diagnosed with skin cancer than all other cancers combined over the past three decades [7].

The management of skin cancer generally involves diagnosis, treatment and follow-up. Melanoma, SCC and BCC are typically detected opportunistically during specific skin examinations by skin cancer specialists or dermatologists, or during general health checks by a general practitioner. In Australia, the standard treatment for primary melanoma is wide local excision of the skin and subcutaneous tissues around the melanoma. The aim is complete surgical excision of all in situ and invasive melanoma components, confirmed by comprehensive histological examination [8]. Surgery is the prime treatment for NMSC: more than 70 % of the BCC lesions recorded in the 2002 National survey were surgically excised [9]. For BCCs not surgically excised, cryotherapy was more commonly used for upper and lower limb lesions than facial lesions, and 10 % of BCCs were treated with curettage and diathermy. Anecdotal evidence suggests that non-surgical treatment has increased since 2002 for superficial BCC with imiquimod in particular. The majority of SCC lesions, regardless of body site, were treated by surgical excision [9, 10]. Confirmation of complete removal of lesions is an essential part of management [10].

The post-treatment follow-up regimen is relatively intensive in Australia with clinical guidelines recommending post melanoma treatment follow-up visits every six-months for five years for patients with stage I disease, three-monthly or four-monthly for five years for patients with stage II or III disease, and yearly thereafter for all patients [8]. NMSC recommendations for follow-up have yet to be established for the detection of further primary tumours, however, some suggestions have been made that six-monthly follow-up for two years may assist in early detection of new primary tumours or of metastatic disease [10].

The incidence of skin cancer increases with age [11, 12]. With most western countries experiencing a demographic transition towards an older cohort, including Australia, the incidence and prevalence of skin cancer is rising, along with the consequential economic impact, even though incidence in younger age groups (i.e., less than 55 years) is stabilising [12–14].

The economic impact of skin cancer can be considered as a combination of direct and indirect costs. Direct costs include the management of skin cancer from diagnosis, treatment to follow-up, and refer to the utilisation of health care resources such as hospital, medical and allied health care services. Indirect costs reflect the lost productivity resulting from an individual’s inability to work (morbidity costs such as sick leave and early retirement) and premature mortality (defined as death before the age of 65 years, the upper limit of the working age in Australia).

The increased incidence and prevalence of skin cancers, combined with the current fiscal environment of limited health care resources and tight budgetary conditions, has increased the importance of understanding the economic impact of skin cancer. From a policy viewpoint, it is important to understand the resource requirements of the current skin cancer burden and the efficiency of competing strategies that are most likely to lower the incidence of skin cancer and, therefore, reduce its burden. Although several international studies have examined the direct cost of skin cancer treatment [15–26], and two have examined indirect costs [27, 28]; our review of the literature found only four studies that had combined both direct and indirect costs in the same analysis [29–32]. Morris et al. [31] estimated the cost of skin cancer in England in 2002 at £240 million, equivalent to £4249 per case (AUD$12,567 in 2010 prices). Tinghog et al. [29] estimated the cost of melanoma and NMSC in Sweden in 2005 at €116 million, equivalent to €3019 per case (AUD$5366 in 2010 prices). Eriksson and Tinghog [32] updated the 2005 Swedish estimate and reported a combined cost of melanoma and NMSC at €136 million. The authors did not report incidence rates or average cost per case, but noted that costs had risen by 14 % after adjusting for inflation since 1995 [32]. O’Dea estimated the cost of skin cancer in New Zealand in 2007 at $NZ123.10 million, equivalent to $NZ1,785 per case (AUD$1650 in 2010 prices) [30]. The general trend in these country specific studies is higher direct costs for NMSC (because of their relatively high prevalence) and higher indirect costs for melanoma (because of its relatively greater severity of illness in those of working age). Although the proportion of costs attributable to indirect costs in all three studies to date appears comparable at around 54 %, total costs appear to vary widely partly due to methodological differences.

New South Wales (NSW) is Australia’s most populated state with an estimated population of 7.29 million (34.5 % of the population of Australia). One of the key objectives of the skin cancer prevention strategy for NSW (2012–2015) is to increase and utilise evidence to inform future planning and development of skin cancer prevention strategies with a priority on: increasing the adoption of UVR protection behaviours; increasing shade provision; and improving polices to increase protection from UVR across a range of settings and life stages [33]. Although an understanding of the magnitude of the economic burden of skin cancer in NSW is important given its high incidence, there is currently a clear lack of such evidence. The objective of this study is to estimate the economic cost (both direct and indirect) of skin cancer in NSW. A lifetime approach is adopted, which estimates costs over the management of skin cancer through diagnosis, treatment and follow-up.

## Methods

### Ethics

Ethics clearance was obtained from the New South Wales Ministry of Health (2012/09/417).

### Economic approach

Economic costs may be estimated using either the prevalence or incidence-based approach to costing. A prevalence-based approach provides estimates of costs for the total population for one year, or costs accumulated over a longer time horizon [34]. An incidence-based approach follows a disease cohort for the duration of the disease and estimates discounted costs [34], it is the most commonly used method as it allows policy makers to understand the potential impact of reducing the incidence of a disease by adopting cost-effective strategies. This analysis uses the incidence based approach to estimate lifetime economic costs. This lifetime perspective reflects the recommended clinical management of skin cancer in Australia [8, 10], managing melanoma and NMSC for five and two years respectively, post diagnosis. Total economic costs in 2010 are derived by multiplying the number of incident cases in 2010 by estimates of the average lifetime direct and indirect costs.

### Epidemiological data

In Australia, melanoma is notifiable to cancer registries. To estimate the incidence of melanoma by age and gender, data were obtained from the NSW Central Cancer Registry (CCR) which at the time of writing was available up to 2008 [35]. These data were combined with Australian Bureau of Statistics (ABS) estimates of the resident NSW population in 2010 [36].

Unlike melanoma and other invasive cancers, NMSC is not notifiable by law to cancer registries in Australia, despite being the most commonly diagnosed cancer. Consequently, rates of NMSC have been estimated from population surveys [5, 9, 37, 38]. The most recent survey was conducted by the National Cancer Control Initiative (NCCI) in 2002 [9]. To estimate the incidence of NMSC by age and gender, data from the NCCI survey were combined with ABS estimates of the resident NSW population in 2010 [36].

### Calculating direct costs of skin cancer

#### Data sources

A range of linked data sources (Table 1) were utilised to determine direct costs: the 45 and Up study (45&Up) [39]; NSW CCR [35]; NSW admitted patient data collection (APDC) [40]; ABS mortality [6]; Pharmaceutical Benefit Schedule (PBS) [41]; Medicare Benefit Schedule (MBS) [42]; and, the NSW Registry of Births, Deaths and Marriages (RBDM) [43].

**Table 1:** Summary of available administrative data

Data source	Abbreviation	Overview of data collected	Period of data collection	Number of possible records
45 and Up survey	45& Up	The 45&Up survey is of individuals aged 45 and over in NSW - the survey includes information on health, lifestyle and other socio-economic factors.	2005-2009	267,119 records (267,119 persons)
NSW Central Cancer Registry	CCR	The CCR includes records for all reported cancer cases from NSW Central Cancer Registry. The CCR records include a range of demographic data items (e.g. date of birth, residential address), staging information, year of diagnosis, plus diagnostic information (e.g. reason for death, Morphology code, Topography Code etc.). The CCR cohort comprises individuals registered on NSW CCR and diagnosed with skin cancer (melanoma, melanoma in situ).	January 1994 – December 2008	63,342 records (60,247 persons)
NSW Admitted Patient Data Collection	APDC	The APDC includes all hospital separations of skin cancer (melanoma, melanoma in situ, non-melanoma) and/or a precursor of skin cancer (sunburn, actinic keratosis or melanocytic naevi) in NSW during the period July 2000-Dec 2011 from all NSW public and private hospitals and day procedure centres. APDC records include a range of demographic data items, administrative items (e.g. admission and separation dates) and diagnostic information (e.g. reason for admission, significant co-morbidities and complications and procedures performed during the admission).	July 2000 – December 2011	406,997 records (256,924 persons)
Australian Bureau of Statistics (ABS) mortality	ABS Mortality	The ABS cohort includes all cases where skin cancer is recorded as the primary or contributing cause of death. Cause of death is coded according to the International Statistical Classification of Diseases and Related Problems (ICD-10-AM).	January 2000 – December 2007	5735 records (5728 persons)
Pharmaceutical Benefit Schedule	PBS	The PBS is administered by Medicare Australia and includes all processing claims and payment benefits for pharmaceutical medications for most medical conditions. This source contains cost and utilisation data by drug for each individual.	2004-2011	35,322,2457 records
Medicare Benefit Schedule	MBS	The MBS is a listing of medical and hospital services that are subsidised by the Australian government. The MBS includes primary care practitioner and specialist consultations and exclude ambulance and allied health services. This source contains costs (including patient out of pocket cost) and utilisation data by service/procedure.	2003-2011	46,012,797 records
NSW Registry of Births, Deaths and Marriages	RBDM deaths	The Registrar of the NSW Registry of Births, Deaths and Marriages (RBDM) is a registry all births and deaths in NSW	RBDM records linked to 45& Up, CCR, APDC and ABS	66,786 records (62,688 persons)

#### Diagnosis of skin cancer

Diagnosis of melanoma (both in-situ and invasive) was determined by the principal diagnoses codes in the Australian modified International Statistical Classification of Diseases and related health problems, 10th revision (ICD-10-AM), specifically codes C43 (malignant melanoma of skin) and D03 (melanoma in situ) [44]. Diagnosis of NMSC was identified using codes C44 (malignant neoplasm of skin) and D04 (carcinoma in situ of skin). Diagnosis was also derived from MBS and PBS utilisation and self-report data from the 45&Up study.

#### Analysis criteria

Costs were calculated for, and compared across, three diagnostic groups: melanoma; NMSC; and, neither melanoma or NMSC. Participants self-reported having either melanoma or NMSC in the 45&Up survey by their responses to the following two questions: “Has a doctor EVER told you that you have melanoma?” “Has a doctor EVER told you that you have skin cancer (not melanoma)?” Self-reported responses were validated by linked records in the CCR, APDC and MBS. Participants without CCR, APDC or MBS records were classified as diagnosis uncertain if their evidence of diagnosis - for either skin cancer - was only self-identification in the 45&Up survey or only relevant MBS records. This group was omitted from analysis due to the ambiguity of their status. The group ‘neither melanoma or NMSC’ had no data relevant to melanoma or NMSC diagnosis and is used as the control group in the regression analysis.

#### Direct costs

Cost groupings were derived from the following sources (data periods appear in brackets):

1. Primary care medical costs: MBS (04/08/2003 to 31/12/2011)
2. Pharmaceutical costs: PBS (01/06/2004 to 31/12/2011)
3. Hospital costs: APDC (01/07/2000 to 31/12/2011)

Variations in data coverage reflect the nature of linking multiple patient-level data sources. To ensure consistency, data from these files were analysed only for their overlap period, 01/06/2004 to 31/12/2011. MBS, PBS and APDC cost estimates were converted to 2010 prices using appropriate price inflators [45].

Estimation of lifetime direct costs used longitudinal methods to estimate average costs (MBS, PBS and APDC), for each diagnosis group, for each calendar year since diagnosis - for 5 years post diagnosis for melanoma and 2 years post diagnosis for NMSC. A cost per annum from year of diagnosis reflects that any cost difference between those with and without a diagnosis is likely to be most pronounced in the first years of diagnosis. Regression analysis is used to estimate the effect on the cost associated with a diagnosis of either melanoma or NMSC, compared to the control group, i.e., those without a diagnosis. The dependent variable is the average annual cost for the year as identified as the year from diagnosis. The covariates included in this model are listed in Table 2 and include the years from diagnosis as an explanatory variable.

**Table 2:** Variables used in costing analysis

Variables	Sources	Question/Variable	Data Format	Analysis summary
Medical Charges	APDC	Average costs per AR-DRGs (from government ‘Costs of Care’ reports	Continuous ($)	Averages and totals across diagnosis groups and periods
	MBS	Charges	Continuous ($)	Averages and totals across diagnosis groups and periods
	PBS	Gross Price	Continuous ($)	Averages and totals across diagnosis groups and periods
Diagnoses Melanoma	CCR	Inclusion in the CCR		Inclusion
	45& Up	Question: ‘Has a doctor ever told you that you have melanoma’?	Yes/No	Proportions
	MBS	Relevant MBS Charges	Continuous ($)	Averages and totals across diagnosis groups and periods
	APDC	Relevant Primary Diagnoses items	Continuous ($)	Averages and totals across diagnosis groups and periods
Diagnosis NMSC	45& Up	Question: ‘Has a doctor ever told you that you have skin cancer (NMSC)’?	Yes/No	Proportions
	MBS	Relevant MBS Charges	Continuous ($)	Averages and totals across diagnosis groups and periods
	APDC	Relevant Primary Diagnoses items	Continuous ($)	Averages and totals across diagnosis groups and periods
Diagnosis Calendar Year (melanoma)	CCR	Year of Diagnosis’ variable	Year	
	APDC	Date of Separation	Year	
	45& Up	Question: ‘At what age were you told you had melanoma?’ (Allows calculation of year with variable ‘Year of Birth’)	Continuous	Averages and totals across diagnosis groups and periods
Diagnosis Calendar Year (NMSC)	45& Up	Question: ‘At what age were you told you had skin cancer (NMSC)’ (Allows calculation of year with variable ‘Year of Birth’)	Continuous	Averages and totals across diagnosis groups and periods
	APDC	Date of Separation	Year	
Demographics	45& Up	Year of birth	Year	Year
		Gender	Female/Male	Proportions
Year of Death	RBDM	Year of death variable	Year	Year

*APDC* NSW Admitted patient data collection; *CCR* NSW Central Cancer Registry; *MBS* Medicate Benefit Schedule; *PBS* Pharmaceutical Benefit Scheme; *RBDM* NSW Registry of Births, Deaths and Marriages; *45&UP* The 45 and Up study

The analysis can be represented by the following equation:

$$ \mathrm{A}\mathrm{L}\mathrm{C} = \mathrm{N}\mathrm{P}\mathrm{V}\left[{\mathrm{CM}}_{\mathrm{i}}\right]\kern0.1em \times \kern0.1em \left.\mathrm{I}\mathrm{M}\right]\kern0.1em +\kern0.10em \mathrm{N}\mathrm{P}\mathrm{V}\left[{\mathrm{CNMSC}}_{\mathrm{i}}\right] \times \mathrm{INMSC} $$

Where:

- ALC = average lifetime cost of skin cancers diagnosed in 2010;
  - ○ NPV = net present value
  - ○ CM_*i*_ = average annual cost per case of melanoma relative to year of diagnosis _*i*_;
  - ○ CNMSC_*i*_ = average annual cost per case of NMSC relative to year of diagnosis _*i*_;
  - ○ IM = incidence of melanoma in 2010;
  - ○ INMSC = incidence of NMSC in 2010.

From each data source (MBS, PBS, APDC) cost data was obtained for each calendar year of the collection period (2004–2011). The data sets were merged on person number and year. The dataset had one record for each participant for each calendar year of the collection period. Records contained fields for calendar year MBS, PBS, APDC costs and, for participants with skin cancer, years since year of diagnosis. Costs are inclusive of patient and government contributions. Participants who died during the collection period only had records for the calendar years in which they were alive. Mortality information was sourced from ABS mortality [6] and the NSW Registry of Births, Deaths and Marriages (RBDM) [43].

Regression modelling for average lifetime direct costs used General Estimating Equations (GEE) analysis. Given a priori evidence of skewed cost data (i.e., large number of zero or small cost observations with a small number of observations with very large costs), GEEs were run with a gamma family and log link. Standard diagnostic tests were conducted (e.g. correlations between independent variables and comparisons of residuals and predicted values). Robust variance estimators were included to ensure more robust estimates of standard errors. Margins (estimates) were derived for average treatment costs for each year from diagnosis by each diagnosis status (melanoma, NMSC) and compared to average annual direct costs for people with no skin cancers. These incremental results were then multiplied by skin cancer incidence figures to derive lifetime direct costs for NSW in 2010.

All regressions were conducted with STATA 12 software. Regression results are available from authors upon request.

### Calculating indirect costs of skin cancer

Indirect costs quantified in this analysis include morbidity and premature mortality for those of working age.

#### Morbidity estimates

The Australian Burden of Disease study provide information on total years lived with a disease and the loss of health (referred to as a disability weight - DW) associated with that disease [46]. Disability weights are based on a scale ranging from 0 to 1 where 0 represents perfect health and 1 represents death [46]. For skin cancer, a range of DWs are used to reflect health states in relation to sequelae. Across the entire disease spectrum, the average DW is 0.19 and 0.06 for melanoma and NMSC, respectively. An estimate of the average health years of life lost due to skin cancer are used as a proxy for morbidity costs in this analysis and are derived by dividing total years lived with skin cancer with the relevant DW.

The human capital approach is used in this analysis to value the loss of productive life. The approach equates the value of a human life to the discounted market value of the output produced by an individual over an expected lifetime. In other words it uses forgone income to estimate forgone productivity [34, 47]. The value of a healthy year of life is equivalent to the average annual earnings in NSW for 2010 - $61,105 for males and $42,238 for females [48]. A further adjustment is made to this value to reflect the likelihood of being employed - 82 % in males and 68 % in females [49].

#### Premature mortality

Premature mortality costs are derived by valuing potential years of life lost (PYLL) due to skin cancer before the age of 65. The most comprehensive source of skin cancer mortality data in NSW is provided by the ABS [6]. Dividing ABS data on PYLL with number of deaths provides an estimate of average years of life lost per death.

ABS data does not, however, report age of death so an alternate means was required to estimate average years of life lost due to skin cancer before the age of 65. The CCR provides individual level data on age at melanoma diagnosis and age at death [35]. For melanoma, CCR data suggest that 38 and 35 % of total years of life were lost in those dying before the age of 65 years, for males and females respectively. In the absence of similar CCR data for NMSC, this proportion is applied to ABS data on PYLL and deaths to estimate the average years of productive life lost per incident case of skin cancer.

As above for morbidity, average annual earnings, adjusted for employment, is used as a proxy for the value of a productive year. All future costs are converted to present value using a 3 % discount rate.

## Results

### Epidemiology

Table 3 provides an overview of skin cancer epidemiology in NSW. In 2010, there were an estimated 3797 new cases of melanoma (2295 male and 1502 female) and 148,610 new cases of NMSC (86,812 male and 61,798 female). Equivalent age-standardised incidence rates are, for melanoma, 65 and 42 per 100,000 males and females, respectively; and, for NMSC, 2449 and 1716 per 100,000 males and females, respectively. For melanoma, the average years of healthy life lost are 3.66 for males (equivalent to 254 days) and 1.77 years for females (equivalent to 123 days). For NMSC, the average years of healthy life lost are 0.05 years for males (equivalent to 16 days) and 0.02 years for females (equivalent to 6 days).

**Table 3:** Epidemiology of skin cancer in New South Wales, 2010

	Melanoma		NMSC	
	Males	Females	Males	Females
Incidence				
Incident cases	2295	1502	86,812^a^	61,798^a^
Incidence rate per 100,000	65	42	2449	1716
Morbidity				
Total years lived with disease	19,332	7089	9933	2533
Average healthy years of life lost due to disease	3.66	1.77	0.05	0.02
Mortality				
Deaths (2010)	359	155	104	41
Total years of life lost	3353	1571	566	104
Average years of life lost	9.34	10.14	5.44	2.53
Mortality before the age of 65 years				
Average years of productive life lost	4.52	5.14	2.07	0.88

^a^NMSC is estimated from a 2002 NCCI report - the incident rate is assumed steady for 2010 and has not risen

In 2010, 359 men and 155 women lost their lives to melanoma corresponding to an age-standardised death rate of 9.8 and 3.6 per 100,000 for males and females, respectively. NMSC claimed the lives of 104 men and 41 females, corresponding to an age-standardised death rate of 2.9 and 0.8 per 100,000 for males and females, respectively. For melanoma, PYLL per death are 9.34 years for males and 10.14 years for females. For NMSC, PYLL per death are 5.44 years for males and 2.53 for females. For those dying of melanoma before the age of 65 years (i.e., 38 % males and 35 % of females), the average years of productive life lost are 4.52 years for males and 5.14 years for females. For those dying of NMSC before the age of 65 years (i.e., 38 % males and 35 % of females), the average years of productive life lost are 2.07 years for males and 0.88 years for females.

### Economic cost of skin cancer

The lifetime economic cost of skin cancer cases in NSW in 2010 is estimated at AUD$536 million or AUD$3514 per incident case (Table 4). Each incident melanoma case costs an average AUD$44,796 compared with AUD$2459 per NMSC case. NMSC costs account for 68 % (AUD$365 million) of total lifetime economic costs. Direct lifetime costs are estimated at AUD$386 million (AUD$2533 per case), with NMSC representing 90 % of total direct costs - AUD$347 million (AUD$2336 per case) and melanoma 10 % of total direct costs - AUD$39 million (AUD$10,230 per case). Indirect lifetime costs are estimated at AUD$150 million (AUD$981 per case) with melanoma representing 88 % of total indirect costs - AUD$131 million (AUD$34,567 per case) and NMSC 12 % of total indirect costs - AUD$18 million (AUD$123 per case). Direct lifetime costs are, on average, higher for females than males with indirect costs, on average, higher for males than females.

**Table 4:** Incident cases, direct cost, indirect cost and total cost of skin cancer in NSW, 2010^a^

	Melanoma	NMSC	Total
Incident cases			
Female	1502	61,798^b^	63,300
Males	2295	86,812^b^	89,106
Total	3797	148,610^b^	152,407
Direct costs			
Females	$16,349,530	$230,717,528	$247,067,059
Cost per female incident case	$10,882	$3733	$3903
Males	$22,494,516	$116,426,574	$138,921,090
Cost per male incident case	$9803	$1341	$1559
Total	$38,844,046	$347,144,102	$385,988,148
Cost per incident case	$10,230	$2336	$2533
Indirect costs			
Females	$34,928,193	$2,259,156	$37,187,349
Cost per female incident case	$23,249	$37	$587
Males	$96,325,255	$16,016,487	$112,341,742
Cost per male incident case	$41,977	$184	$1261
Total	$131,253,448	$18,275,643	$149,529,091
Cost per incident case	$34,567	$123	$981
Total costs	$170,097,494	$365,419,746	$535,517,240
Cost per incident case	$44,796	$2459	$3514

^a^Average cost may not equate to total cost divided with cases due to rounding

^b^NMSC is estimated from a 2002 NCCI report - the incident rate is assumed steady for 2010 and has not risen

## Discussion

In conducting this study a range of data sources and methods were used. As such, a number of potential limitations and strengths of the analysis need to be considered.

### Limitations

First, while the CCR data informed the incidence of melanoma in NSW, partial data sources were relied on to develop an understanding of the incidence of NMSC. These data sources are dated and may not reflect recent changes in incidence of NMSC. Second, our analysis did not consider the cost of skin cancer by stage of diagnosis, type of treatment, treatment provider or socio-economic status. Evidence suggests that there are variations in costs across these categories [15–18]. In our study, although CCR provided information on staging of disease at time of diagnosis, no other data set had comparable data. Third, the base year for the analysis is 2010. This year is appropriate given data availability but it is acknowledged that skin cancer management may have changed over recent years. Fourth, a limitation of productivity estimates is a lack of complete Industry data. A recent study conducted by Safe Work Australia on exposure to direct sunlight and the provision of sun exposure controls in Australian workplaces, provides evidence that certain workers have a higher likelihood of being exposed to direct sunlight [50]. Further, the report suggests that the provision of sun protection (i.e. sunscreen, protective clothing, hats, sunglasses and being able to reorganise work outside peak UVR hours) was affected by worker employment and demographic characteristics [50]. The lack of data precludes a more robust assessment of Industry-related costs due to skin cancer in this analysis. Fifth, the analysis did not value the contribution made by carers. A report by Access Economics for Carer’s Australia examined and valued the amount of informal care being provided in Australia [51]. The report suggests that in 2010, over 1 in 8 Australians (2.87 million people) were estimated to be providing informal care with each carer providing up to 9 h per week in informal care. In the absence of any data related to the number of carers’ for skin cancer patients, carer costs were excluded.

### Strengths

A strength of the analysis is the use of linked epidemiological data. Data linkage transforms routinely collected administrative data into a powerful resource for research. For the current study, the linking of administrative data provided a rich source of complementary information on: diagnosis of melanoma (CCR); costs associated with skin cancer (MBS, PBS and the APDC); and skin cancer mortality (CCR, APDC). Second, maximising the utility of the linked data set required a flexible data analysis that was logical and robust. The linkage process followed a sequential strategy that aimed to minimise the number of false positive records. The diagnosis criteria for melanoma and NMSC were informed using relevant international classification of disease coding. The statistical approach enabled a comprehensive and rigorous assessment of the lifetime costs of skin cancer in NSW. The costing approach was broadly consistent with other studies analysing Medicare-linked population-based databases in the United States [15–18, 52]. Third, consistent with other costing studies, our analysis has attempted to place a monetary value on indirect costs [27–29, 53]. Our analysis only considers the economic value of productive years of life lost is a conservative estimate of mortality. Other studies quantify the economic value of all years of life lost, not just that before the age of 65 years [28].

### Comparability with other studies

In spite of methodological differences, the estimated cost of skin cancer in NSW is generally consistent with previous Australian and International studies (Table 5) [29–31, 54, 55]. Compared with our estimate of the average cost per incident case of melanoma (AUD$44,796), the English study calculated AUD$67,567 [31], the Swedish study AUD$66,738 [29], and the New Zealand study AUD$30,326 [30]. Compared with our estimate of the average cost per incident case of NMSC (AUD$2459), the English study calculated AUD$5955 [31], the Swedish study AUD$1775 [29], and the New Zealand study AUD$802 [30].

**Table 5:** Summary of skin cancer costing studies, Australia, England, Sweden and New Zealand

Study	Country	Year and currency	Cases	Direct cost (million)	Indirect costs (million)	Total cost (million)	Cost per case	Equivalent AUD $2010
Morris	England	2002 UK pounds						
Melanoma			6062	£24	£114	£138	£22,835	$67,537
NMSC			50,394	£97	£5	£101	£2014	$5955
Skin cancer			56,456	£121	£119	£240	£4249	$12,567
Tinghog	Sweden	2005 Euros						
Melanoma			2122	€ 22	€ 58	€ 80	€ 37,545	$66,738
NMSC			36,262	€ 31	€ 5	€ 36	€ 998	$1775
Skin cancer			38,384	€ 53	€ 63	€ 116	€ 3019	$5366
O’Dea	New Zealand	2007/8 NZ dollar						
Melanoma			1982	$6	$59	$65	$32,795	$30,326
NMSC			67,000	$51	$7	$58	$867	$802
Skin cancer			68,982	$57	$66	$123	$1785	$1650
AIHW	Australia	1993-94 AUD						
Melanoma			6954	$66		$66	$9433	$14,805
NMSC			243,691	$232		$232	$953	$1496
Skin cancer			250,645	$298		$298	$1189	$1865
AIHW	Australia	2000-01 AUD						
Melanoma			8885	$30		$30	$3376	$4561
NMSC			364,140	$264		$264	$725	$979
Skin cancer			373,025	$294		$294	$788	$1065

Source: Morris et al. (2009) [31], Tinghog et al. (2008) [29], O’Dea (2009) [30]

Previous estimates for the cost of skin cancer in Australia only valued direct costs. Compared with our estimate of the average direct cost per incident case of melanoma (AUD$10,230), other Australian estimates ranged from AUD$14,805 in a 1993–94 study [55] to AUD$4561 in a 2000–01 study [54]. Compared with our estimate of the average direct cost per incident case NMSC (AUD$2336), other Australian estimates ranged from AUD$1496 for the 1993–94 study [55] and AUD$979 for the 2000–01 study [54].

## Conclusion

This study provides new evidence on the economic costs associated with skin cancer in NSW, Australia. Although this analysis is based on the latest epidemiological and economic evidence, there are still large knowledge gaps in understanding the wider impact of skin cancer on society. This lack of data means that the study most likely under-estimates the true cost of skin cancer in NSW.

A key factor underpinning the strategic plan of the Cancer Institute NSW is the knowledge that both melanoma and NMSC are highly preventable. The most effective means of reducing risk of developing skin cancer is to avoid direct exposure to UVR during the time of day when solar UVR levels are moderate to extreme. As a consequence of this analysis, we are in a better position to quantify the savings to society of reducing the incidence of skin cancer through preventive strategies such as sunscreen or mass media campaigns efforts. These savings are likely to be significant given the average lifetime cost of skin cancer in NSW is AUD$44,796 per melanoma case and AUD$2459 per NMSC case.

## Abbreviations

APDC: Admitted patient data collection
AUD: Australian dollar
BCC: Basal cell carcinoma
CCR: Central Cancer Registry
DW: Disability weights
GEE: General estimating equations
ICD-10-AM: International Statistical Classification of Diseases, 10th revision
MBS: Medical Benefits Scheme
NZ: New Zealand
NMSC: Non-melanoma skin cancer
NSW: New South Wales
PBS: Pharmaceutical Benefits Scheme
PYLL: Potential years of life lost
SCC: Squamous cell carcinoma
UK: United Kingdom
UVR: Ultra violet radiation
